# 帕博利珠单抗联合安罗替尼四线治疗*EGFR*基因敏感突变阳性肺腺癌1例

**DOI:** 10.3779/j.issn.1009-3419.2021.102.25

**Published:** 2021-10-20

**Authors:** 莉玲 黄, 燕 秦, 凤仪 赵, 生余 周, 远凯 石

**Affiliations:** 100021 北京，国家癌症中心/国家肿瘤临床医学研究中心/中国医学科学院肿瘤医院内科/国家抗肿瘤药物临床研究北京市重点实验室 Department of Medical Oncology, National Cancer Center/National Clinical Research Center for Cancer/Cancer Hospital, Chinese Academy of Medical Sciences & Peking Union Medical College, Beijing Key Laboratory of Clinical Study on Anticancer Molecular Targeted Drugs, Beijing 100021, China

**Keywords:** 肺肿瘤, 帕博利珠单抗, 安罗替尼, 免疫治疗, 抗血管生成, Lung neoplasms, Pembrolizumab, Anlotinib, Immunotherapy, Anti-angiogenesis

## Abstract

中国晚期肺腺癌表皮生长因子受体（epidermal growth factor receptor, *EGFR*）基因敏感突变比例约为45.7%。*EGFR*基因敏感突变阳性的晚期非小细胞肺癌（non-small cell lung cancer, NSCLC）患者在EGFR-酪氨酸激酶抑制剂（tyrosine kinase inhibitor, TKI）治疗和化疗失败后治疗选择有限，寻找有效的治疗方案是临床的迫切需求。我们报道1例82岁的*EGFR*基因敏感突变阳性晚期肺腺癌女性患者，在奥希替尼、化疗及安罗替尼单药治疗均失败后，通过帕博利珠单抗和安罗替尼的联合治疗，截至2021年1月12日已获得超过21个月的无进展生存期，耐受性良好。

## Abstract (en)

45.7% of Chinese patients with advanced lung adenocarcinoma were reported to harbour sensitizing epidermal growth factor receptor (*EGFR*) mutations. Limited therapeutic options are left for non-small cell lung cancer (NSCLC) harbouring sensitizing *EGFR* mutations after failure of EGFR-tyrosine kinase inhibitor (TKI) therapy and chemotherapy, finding effective options for them is an unmet clinic need. Herein we reported a case that till January 12, 2021, an 82-year-old female with sensitizing *EGFR*-mutant advanced lung adenocarcinoma received a surprising progression-free survival (PFS) benefit of over 21 months from the combination therapy of pembrolizumab and anlotinib after her failure of treatments of osimertinib, chemotherapy and anlotinib-monotherapy.

## 1 病例资料

患者，女，82岁，因“确诊右肺腺癌伴肾上腺转移10月余，一线奥希替尼治疗后进展”于2018年11月1日就诊于中国医学科学院肿瘤医院内科。患者于2017年12月18日因刺激性呛咳就诊于美国麻省总医院，经病理和影像检查确诊为右肺低分化腺癌IV期伴肾上腺转移，肿瘤组织基因检测结果显示表皮生长因子受体（epidermal growth factor receptor, *EGFR*）21号外显子L858R基因突变阳性，免疫组织化学染色结果显示甲状腺转录因子1（thyroid transcription factor-1, TTF-1）（-），Napsin A（-），p40（-），间变性淋巴瘤激酶（anaplastic lymphoma kinase, ALK）（-），c-ros肉瘤致癌因子-受体酪氨酸激酶（ROS proto-oncogene 1, receptor tyrosine kinase, ROS1）（-），程序性死亡受体配体1（programmed cell death-ligand 1, PD-L1）肿瘤比例评分（tumor proportion score, TPS）为2%，CD8^+^肿瘤浸润淋巴细胞很少且分散，占 < 5%肿瘤细胞。2017年12月底开始接受一线治疗：第三代EGFR酪氨酸激酶抑制剂（tyrosine kinase inhibitor, TKI）甲磺酸奥希替尼片80 mg每天一次口服，最佳疗效评价为部分缓解（partial response, PR），但2018年6月复查胸部计算机断层扫描（computed tomography, CT）提示疾病进展（progressive disease, PD），患者再次于麻省总医院行肺部肿瘤组织活检，病理诊断为腺癌伴鳞状细胞癌分化，第二次基因检测结果同样显示*EGFR* 21号外显子L858R突变阳性。患者继续接受奥希替尼治疗，直到2018年11月1日就诊于我院，2018年11月2日胸腹部CT检查结果示右上肺叶病变为7.2 cm×3.8 cm，伴有胸膜和肾上腺转移，疗效评价为PD。既往史：血压偏高，间断应用降压药治疗。查体：右肺呼吸音粗，全身浅表淋巴结未见肿大。入院前血常规生化检查未见异常。

2018年11月16日患者接受二线治疗：培美曲塞单药化疗（500 mg，静脉滴注，第1天，每3周为1个周期），2个周期后CT检查评价疗效为疾病稳定（stable disease, SD）：右肺上叶不规则肿物部分层面较前略缩小，部分较前饱满，最大截面为7.2 cm×4.0 cm。患者诉不能耐受培美曲塞，表现为III度乏力，2个周期后即停用该药。2019年1月30日患者接受三线治疗：盐酸安罗替尼胶囊8 mg隔日一次口服，d1-d14，每3周为1个周期，2个周期后复查肺部原发病灶较前稍增大，最大截面为8.2 cm×4.2 cm，疗效评价为疾病稳定（stable disease, SD）。患者及家属拒绝后续放化疗，结合初诊时PD-L1 TPS为2%的状态和患者意愿，2019年4月3日患者开始四线帕博利珠单抗联合安罗替尼方案治疗。因患者高龄、既往药物治疗耐受性较差，帕博利珠单抗剂量减半为100 mg静脉滴注，每3周1次，安罗替尼剂量同前。4个周期治疗后复查CT发现肺部病灶内实性成分明显减少，病灶内部新见空洞影，疗效评价为PR。10个周期后复查CT，肺部病灶3.7 cm×3.1 cm，胸膜和肾上腺病灶维持稳定，疗效评价为持续PR，患者对此联合方案的耐受良好。受新冠肺炎疫情影响，帕博利珠单抗自2020年1月起延长至100 mg每4-6周输注1次；安罗替尼剂量和周期维持不变。末次CT影像随访时间为2020年12月3日，肺部病灶3.6 cm×3.1 cm，胸膜和肾上腺病灶同前相仿，疗效评价为持续PR。末次电话随访时间为2021年1月12日，患者已接受了18次帕博利珠单抗和28个周期安罗替尼治疗，无进展生存期（progression-free survival, PFS）超过21个月（图 1）。患者对该方案耐受性良好，主要发生1级-2级治疗相关不良事件，包括皮疹、疲劳、厌食、咳嗽。

**1:**
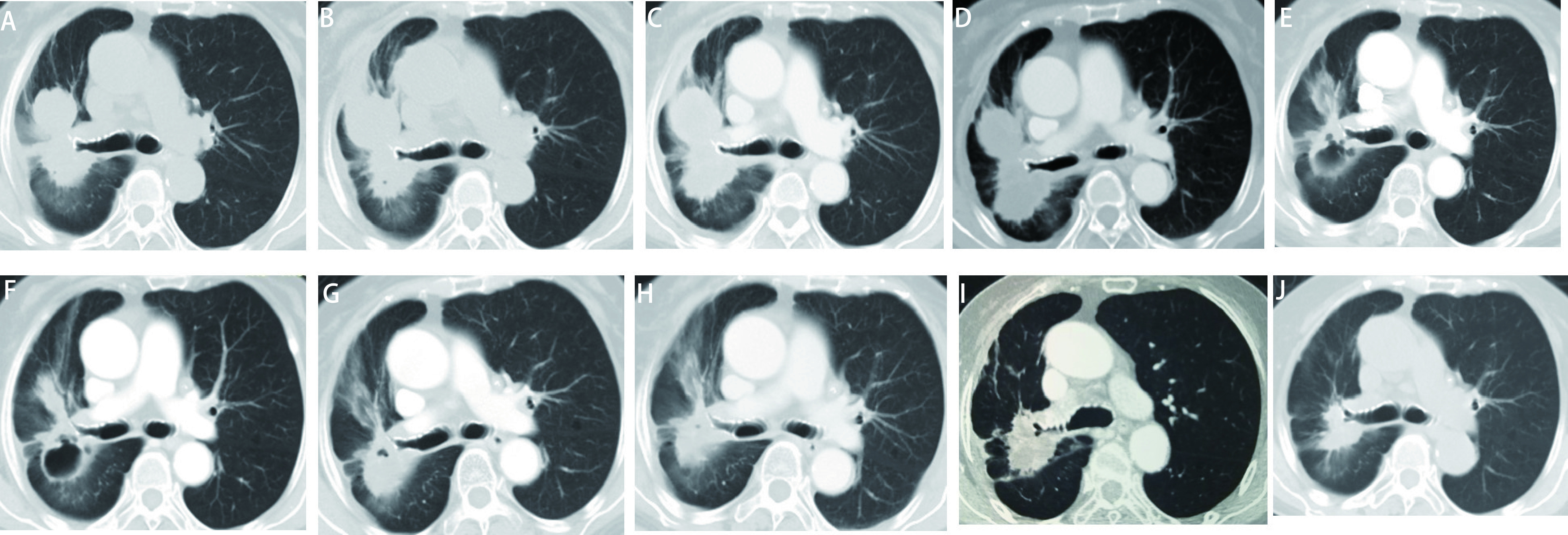
CT显示患者接受四线帕博利珠单抗和安罗替尼联合治疗有效。A：患者一线奥希替尼治疗10月余首次就诊于我院, 病灶7.2 cm×3.8 cm；B：二线培美曲塞单药治疗2个周期后, 病灶7.2 cm×4.0 cm，疗效评价为疾病稳定（stable disease, SD）；C：三线安罗替尼单药治疗2个周期后，病灶8.2 cm×4.2 cm，疗效评价为SD；D：四线帕博利珠单抗和安罗替尼联合治疗2个周期后，病灶7.4 cm×4.2 cm, 疗效评价为SD；E：四线帕博利珠单抗和安罗替尼联合治疗4个周期后，肺部病灶内实性成分明显减少，病灶内部新见空洞影，疗效评价为部分缓解（partial response, PR）；F：四线帕博利珠单抗和安罗替尼联合治疗6个周期后，实性成分较前轻微减少，病灶6.6 cm×4.6 cm，疗效评价为持续PR；G：四线帕博利珠单抗和安罗替尼联合治疗8个周期后，之前的肺部空洞被液体填充；H：四线帕博利珠单抗和安罗替尼联合治疗10个周期后，CT检查时间为2019年12月30日，病灶3.7 cm×3.1 cm，疗效评价为持续PR。受新冠疫情影响，自2020年1月起帕博利珠单抗延长至100 mg每4-6周输注1次；安罗替尼仍为8 mg，隔日一次口服，第1-14天，每3周为1个周期；I：CT检查时间为2020年7月28日，病灶稳定，疗效评价为持续PR；J：末次CT检查随访时间为2020年12月3日，病灶3.6 cm×3.1 cm，疗效评价为持续PR。 Computed tomography (CT) scans showed the clinical response to fourth-line combination therapy of pembrolizumab and anlotinib. A: First presentation at our hospital after over 10 months of osimertinib, the primary pulmonary lesion was 7.2 cm×3.8 cm; B: After 2 cycles of second-line therapy of pemetrexed, the lesion was 7.2 cm×4.0 cm, response evaluation was stable disease (SD); C: After 2 cycles of third-line therapy of anlotinib, the lesion was 8.2 cm×4.2 cm, response evaluation was SD; D: After 2 cycles of fourth-line therapy of pembrolizumab plus anlotinib, the lesion was 7.4 cm×4.2 cm, response evaluation was SD; E: After 4 cycles of fourth-line therapy of pembrolizumab plus anlotinib, the solid components reduced remarkably and the cavity formed, response evaluation was partial response (PR); F: After 6 cycles of fourth-line therapy of pembrolizumab plus anlotinib, solid components decreased slightly, the lesion was 6.6 cm×4.6 cm, response evaluation was persistent PR; G: After 8 cycles of fourth-line therapy of pembrolizumab plus anlotinib, the former pulmonary cavity was filled with liquid; H: After 10 cycles of fourth-line therapy of pembrolizumab plus anlotinib, the CT was performed on December 30, 2019, the lesion was 3.7 cm×3.1 cm, response evaluation was persistent PR. Due to the influence of COVID-19's epidemic situation, since January 2020 the administration of pembrolizumab prolonged to 100 mg *ivgtt* d1, *q4-6w*, while anlotinib remained 8 mg *po qod* d1-d14, *q3w*; I: The CT was performed on July 28, 2020, the lesion was stable, response evaluation was persistent PR; J: The lastest follow-up of CT scan was on December 3, 2020, the lesion was 3.6 cm×3.1 cm, response evaluation was persistent PR.

## 2 讨论

中国晚期肺腺癌患者*EGFR*基因敏感突变比例约为45.7%^[1]^。*EGFR*基因敏感突变阳性的晚期非小细胞肺癌（non-small cell lung cancer, NSCLC）患者在EGFR-TKI治疗和化疗失败后治疗选择有限，寻找有效治疗方案是临床的迫切需求。

本文报告了1例奥希替尼、培美曲塞、安罗替尼单药治疗失败的晚期*EGFR*基因敏感突变阳性老年女性肺腺癌患者，经帕博利珠单抗联合安罗替尼方案的四线治疗，获得持久的PFS并且耐受性良好，值得未来进行更深入的研究。

安罗替尼是一种多靶点TKI，针对多个血管生成和增殖途径发挥作用，已被国家食品药品监督管理局批准用于治疗既往至少接受过2种系统化疗后出现进展或复发的局部晚期或转移性NSCLC患者^[2]^，但该患者安罗替尼三线单药治疗效果不佳。帕博利珠单抗是一种抗程序性细胞死亡-1（programmed cell death 1, PD-1）单克隆抗体，已被美国食品药品监督管理局批准单药用于在含铂化疗期间或之后进展或转移的PD-L1 TPS≥1%的NSCLC患者的治疗^[3]^。值得注意的是，由于*EGFR*基因敏感突变的存在，帕博利珠单抗的有效性急剧降低。Keynote-010研究^[3]^结果表明，PD-L1 TPS ≥1%的晚期NSCLC患者接受帕博利珠单抗2 mg/kg治疗后的PFS为5.0个月，亚组分析中*EGFR*基因敏感突变亚组比野生型亚组从帕博利珠单抗的获益更低。鉴于这例患者PD-L1的TPS只表达2%，加上其*EGFR*基因敏感阳性突变状态，我们有理由推测帕博利珠单抗单药不太可能给该患者带来如此好的生存获益，另外该患者CT检查显示帕博利珠单抗联合安罗替尼治疗后出现的空洞是典型抗血管生成药物有效的表现，提示该患者的治疗效果是得益于帕博利珠单抗和安罗替尼的联合应用。

近年来，抗血管生成药物联合免疫检查点抑制剂产生的协同抗肿瘤效应得到了多项研究结果的支持。临床前研究^[4]^发现，抗血管生成药物可促进肿瘤血管正常化，并从多方面调节免疫微环境，进而激活免疫系统，包括促进树突状细胞成熟、恢复T细胞动员和浸润、淋巴细胞黏附以及减少抑制性免疫细胞的诱导和增殖。同时，多种先天性免疫细胞和获得性免疫细胞参与了肿瘤血管的形成，免疫检查点抑制剂进行的免疫治疗能促进肿瘤血管正常化^[5]^。这一联合策略已在肾癌、肝癌等肿瘤的大型临床试验中显现了显著的疗效^[6, 7]^。在NSCLC，Impower 150研究^[8]^是一次成功的尝试，该研究对比了贝伐珠单抗、卡铂、紫杉醇联合阿替利珠单抗方案（ABCP）和贝伐珠单抗、卡铂、紫杉醇（BCP）方案一线治疗转移性非鳞状NSCLC患者的疗效和安全性，结果表明ABCP组患者的PFS和总生存（overall survival, OS）显著延长。亚组分析结果显示，在*EGFR*基因突变或*ALK*融合基因阳性的患者中，ABCP组比BCP组显示了更好的生存获益（PFS：9.7个月 *vs* 6.1个月，HR=0.59，95%CI：0.37-0.94）。另一项III期临床试验^[9]^比较了贝伐珠单抗、卡铂、紫杉醇联合或不联合纳武利尤单抗一线治疗晚期或复发性非鳞状NSCLC患者的疗效，初步结果显示联合纳武利尤单抗组显著延长患者的PFS（12.12个月 *vs* 8.11个月，HR=0.56，*P* < 0.000, 1）。一项Ib期研究^[10]^探索了信迪利单抗联合安罗替尼一线治疗无*EGFR*基因敏感突变、无*ALK* /*ROS1*融合基因阳性的22例局部晚期或转移性NSCLC患者，客观反应率为72.7%，中位PFS为15个月。

抗血管生成和免疫治疗的联合应用已显现出初步疗效，但仍有很多问题值得深入探讨。基于已有的研究结果，目前较被认可的免疫治疗疗效标志物PD-L1、肿瘤突变负荷（tumor mutation burden, TMB）在联合治疗中预测价值有限，未来应继续探索合适的疗效预测生物标志物及基因突变谱以筛选更适宜的联合治疗人群^[11]^。不同剂量的抗血管生成药物可能对患者免疫功能的影响截然不同，Lin等^[12]^的研究发现，低剂量抗血管生成药物治疗可能通过增强巨噬细胞的M1极化和增强CD8^+^ T细胞功能发挥免疫促进作用，而高剂量抗血管生成药物治疗可能导致微环境的免疫抑制，因此抗血管生成药物的合适剂量是抗血管生成-免疫双药联合方案需要考虑的问题。
